# A Monopole and Dipole Hybrid Antenna Array for Human Brain Imaging at 10.5 Tesla

**DOI:** 10.1109/lawp.2022.3183206

**Published:** 2022-06-15

**Authors:** Myung Kyun Woo, Lance DelaBarre, Matt Waks, Russell Lagore, Jerahmie Radder, Steve Jungst, Chang-Ki Kang, Kamil Ugurbil, Gregor Adriany

**Affiliations:** Department of Biomedical Engineering, School of Electrical Engineering, University of Ulsan, Ulsan 44005, South Korea; Center for Magnetic Resonance Research (CMRR), University of Minnesota, Minneapolis, MN 55455 USA; Center for Magnetic Resonance Research (CMRR), University of Minnesota, Minneapolis, MN 55455 USA; Center for Magnetic Resonance Research (CMRR), University of Minnesota, Minneapolis, MN 55455 USA; Center for Magnetic Resonance Research (CMRR), University of Minnesota, Minneapolis, MN 55455 USA; Center for Magnetic Resonance Research (CMRR), University of Minnesota, Minneapolis, MN 55455 USA; Department of Radiological Science, College of Health Science, Gachon University, Incheon 1342, South Korea; Center for Magnetic Resonance Research, University of Minnesota, Minneapolis, MN 55455 USA; Center for Magnetic Resonance Research (CMRR), University of Minnesota, Minneapolis, MN 55455 USA

**Keywords:** Dipole antenna, human brain imaging, monopole antenna, multi-channel array, ultra-high field imaging

## Abstract

In this letter, we evaluate antenna designs for ultra-high frequency and field (UHF) human brain magnetic resonance imaging (MRI) at 10.5 tesla (T). Although MRI at such UHF is expected to provide major signal-to-noise gains, the frequency of interest, 447 MHz, presents us with challenges regarding improved B_1_^+^ efficiency, image homogeneity, specific absorption rate (SAR), and antenna element decoupling for array configurations. To address these challenges, we propose the use of both monopole and dipole antennas in a novel hybrid configuration, which we refer to as a mono-dipole hybrid antenna (MDH) array. Compared to an 8-channel dipole antenna array of the same dimensions, the 8-channel MDH array showed an improvement in decoupling between adjacent array channels, as well as ~18% higher B_1_^+^ and SAR efficiency near the central region of the phantom based on simulation and experiment. However, the performances of the MDH and dipole antenna arrays were overall similar when evaluating a human model in terms of peak B_1_^+^ efficiency, 10 g SAR, and SAR efficiency. Finally, the concept of an MDH array showed an advantage in improved decoupling, SAR, and B_1_^+^ near the superior region of the brain for human brain imaging.

## I. Introduction

**Fig. 1.**
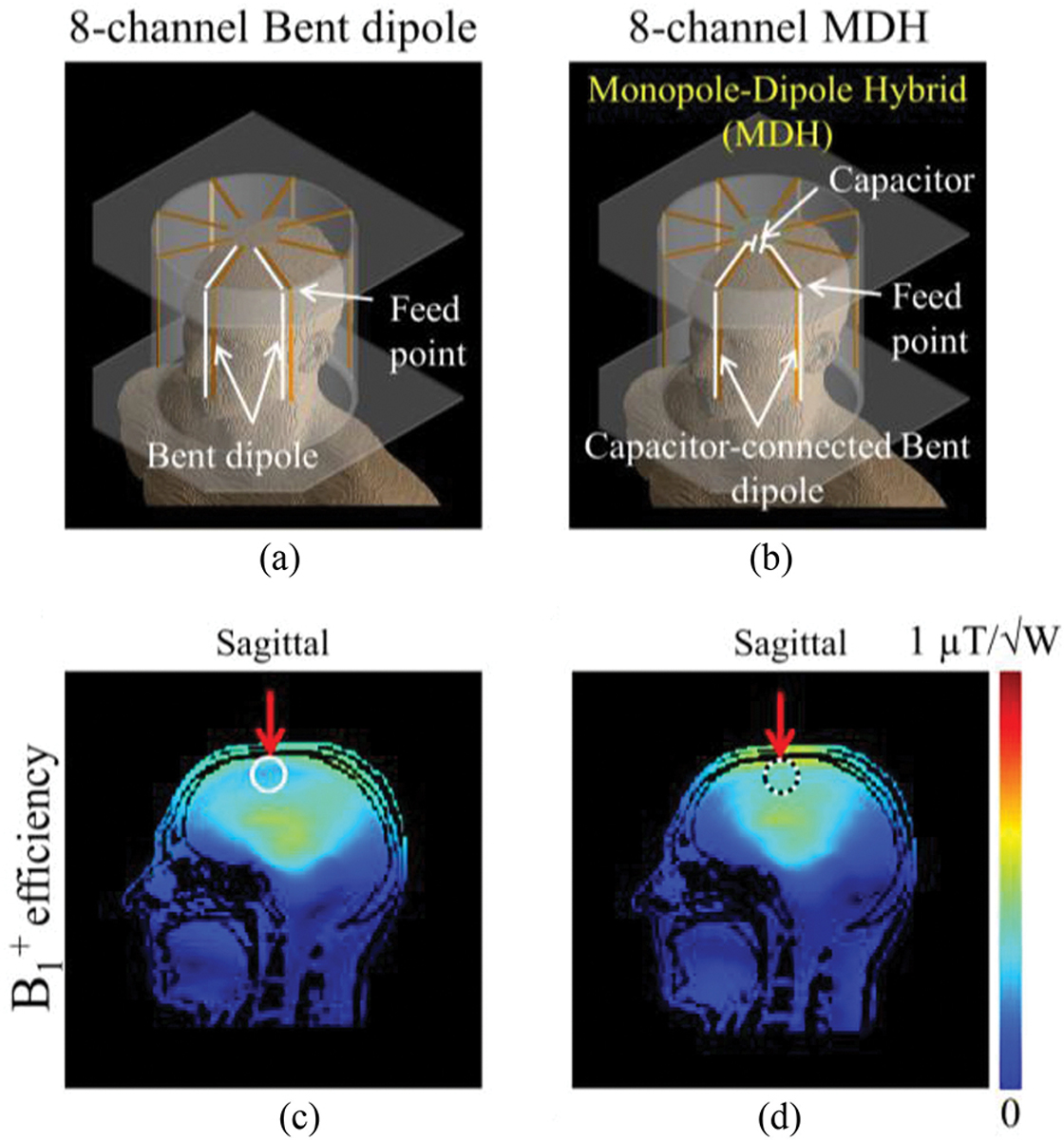
3-D drawing of the 8-channel bent dipole (a) and MDH (b) arrays with a human model. Sagittal B_1_^+^ fields obtained with the 8-channel bent dipole antenna (c) and MDH (d) arrays. Significant B_1_^+^ improvement was shown for the superior part of the brain.

Radiative antenna type arrays [1]–[7] have been advantageously used at ultrahigh frequency and fields (UHF) [8]–[12] for magnetic resonance imaging (MRI) in attempts to overcome wavelengths effects in the human body. At the associated UHF operating frequencies, arrays consisting of λ/2 dipole antennas have been particularly successful and have demonstrated sufficiently uniform magnetic (B) field distributions and improved penetration compared to the more traditional loop-type [3], [5], [13]–[16] radio frequency (RF) coils. For optimal use of dipoles in whole brain imaging and to achieve higher B_1_^+^ efficiency (defined as the B_1_ amplitude per unit square root of total power), classical straight dipole antenna arrays can be modified to better follow the contours and shape of the human head. To achieve this, bent/folded dipole antenna arrays have been previously presented and evaluated [17]–[20]. In a typical bent dipole antenna array design, one leg of the individual dipole antenna element is bent inwards which effectively results in higher conductor density at the top of the brain [Fig. 1(a)]. However, a multichannel array comprised of these elements cannot generate sufficient B_1_^+^ fields at the superior part of the brain for whole brain imaging, as shown in Fig. 1(c). Alternatively, a floating RF ground endplate and the use of dielectric material have been proposed to improve B-field homogeneity in the upper part of the head for birdcage type resonators [21], [22].

For UHF head applications at 7 tesla (T), monopole antenna arrays with λ/4 conductors connected to a common ground plane elegantly incorporate an RF ground endplate into the resonance structure and have been suggested as an alternative to the physically longer dipole antennas [23]. These monopole antenna arrays showed enhanced B_1_^+^ efficiency compared to loop and dipole antenna arrays [6], [23], [24]. However, while monopole antenna arrays have been used to generate an efficient B-field in the superior part of the brain, they also showed a significant electric (E)-field hot spot in that area with related consequences for specific absorption rate (SAR).

Here, we attempt to address this and compare a compromise between an 8-channel bent dipole [Fig. 1(a)] and monopole-dipole hybrid antenna (MDH) array [Fig. 1(b)] and propose this new array for UHF brain MRI applications. Compared to the bent dipole antenna array [Fig. 1(c)], B_1_^+^ field improvement with this MDH array [Fig. 1(d)] was observed in the superior part of the brain. By capacitively connecting the bent poles of the dipole antenna legs, the MDH array also reduced an SAR hot spot that is present with monopole antenna arrays [24] that utilize a solid ground plane.

To further investigate the use of this MDH array as an RF transmitter at 10.5 T, we compared it to an 8-channel end-loaded dipole antenna array of similar inner dimensions. Scattering (S) parameters of the arrays were measured and compared with a cylindrical phantom on the bench, and noise covariance maps were acquired with a 10.5 T MRI scanner. The B_1_^+^ efficiency was simulated and the results were compared to data obtained in MR experiments. For safety validation, 10 g SAR and SAR efficiency [defined as B_1_^+^/√(peak 10 g SAR)] were calculated. For human brain imaging, B_1_^+^ efficiency, 10 g SAR, and SAR efficiency of the arrays were simulated with a human model (Duke) and compared [25].

## II. Methods

### A. Construction of 8-Channel Arrays

**Fig. 2.**
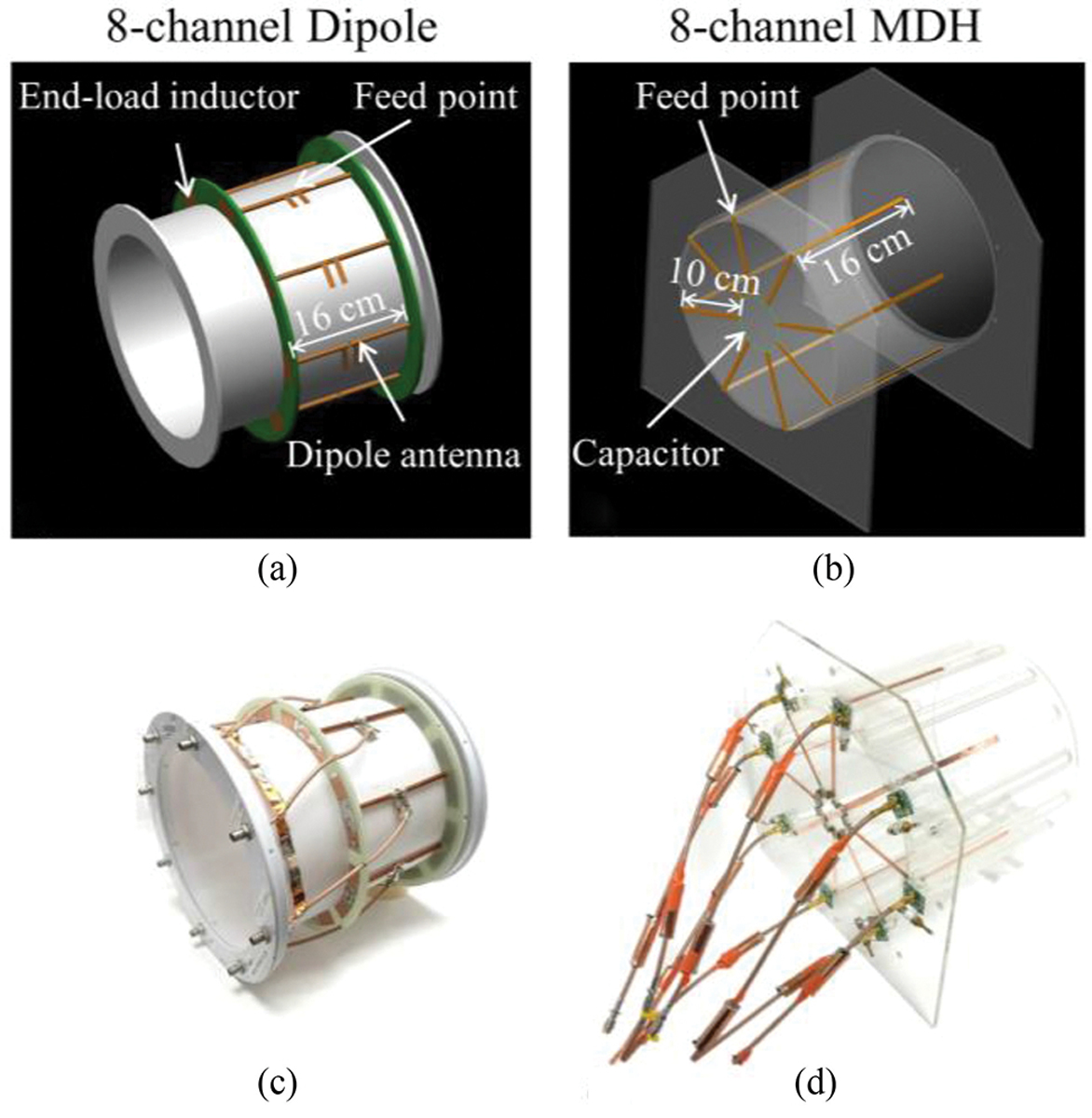
3-D modeling (a) and (b) and photographs (c) and (d) of home-built eight-channel end-loaded dipole and MDH arrays.

An 8-channel dipole antenna array was three-dimensional (3-D) modeled and the housing was fabricated in-house using a fused deposition modeling 3-D printer (F410, Fusion3 Design, Greensboro, NC, USA) as shown in Fig. 2(a) and (c). As shown in Fig. 2(b) and (d), an 8-channel MDH array was built on an acrylic former. Each array was built onto a cylindrical former with the same inner diameter of 25 cm and a length of 20 cm. The spacing between the neighboring elements of the 8-channel dipole and MDH arrays was ~9 cm without any decoupling circuitry.

To accommodate realistic human head dimensions, the individual dipoles of the dipole antenna array were shortened to 16 cm by use of end-loaded inductors [26]. An additional common ground ring was created using 680 pF capacitors (100E series, American Technical Ceramics, Huntington Station, NY, USA), thus eliminating the need for cable traps beyond the housing, as indicated shown in Fig. 2(c). As shown in Fig. 2(b) and (d), the MDH array also has eight equally spaced dipole antennas, and the length of one the legs of each dipole antenna was 16 cm and the other was effectively shortened to 10 cm with a common ground using 330 pF capacitors. By connecting one pole of each dipole, an effective radial ground plane was created. Floating cable traps [27], [28] were also utilized in each coaxial feedline in the MDH array to suppress sheath currents.

A 16-channel vector network analyzer (ZNBT8, ROHDE & SCHWARZ, Munich, Germany) was used for all bench measurements. S_11_ (reflection coefficient) and *S*_21_ (coupling coefficient with adjacent channels) were measured when all arrays were loaded with a cylindrical phantom.

### B. Simulation and Numerical Analysis

To evaluate the B_1_^+^ efficiency, the B_1_ transmits fields were normalized to the net input power. Simulated B_1_^+^ efficiency, 10 g SAR, and SAR efficiency were calculated by electromagnetic simulation (XFdtd, REMCOM, State College, PA). All data were post-calculated with MATLAB (The Mathworks, Inc., Natick, MA, USA). B_1_^+^ fields were determined as $B_{1}^{+}=\left|\frac{B_{x}+iB_{y}}{2}\right|$, where B_x_ and B_y_ were the complex amplitudes of x- and y-oriented RF magnetic fields, respectively [29]. For safety validation, 10 g SAR values for each array were calculated with the E-field and SAR efficiency values were then compared between the arrays. For a quantitative comparison, the highest B_1_^+^ efficiency, 10 g SAR, and SAR efficiency areas were selected as regions of interest (ROI) for one area in the axial and coronal planes of each array. The values for ROI (2 mm isotropic voxel) are indicated below each coronal figure. The B_1_^+^ efficiency, 10 g SAR, and SAR efficiency of the arrays with the human model were calculated and compared in the axial, coronal, and sagittal planes.

### C. Experiments With the Phantom

All phantom experiments were performed using a 10.5 T / 88 cm whole-body magnet (Agilent, Santa Clara, CA) fitted with a SC72 body gradient coil in conjunction with a 16-channel parallel transmit (pTx) system (Siemens Healthineers, Erlangen, Germany).

The size of the cylindrical phantom (a solution of sucrose and NaCl in distilled water: *ε*_r_ = 49 and *σ* = 0.6 S/m) used for the comparison of the arrays was 18 cm in diameter and 30.5 cm in height [30]. As an experimental validation of the achievable decoupling, noise covariance matrices of the 8-channel dipole and MDH arrays were acquired to evaluate the crosstalk between all elements [31]–[34]. An actual flip angle imaging sequence (TR_1_/TR_2_ = 25/115 ms, TE = 3.39 ms, nominal flip angle = 60°, GRAPPA (R = 2), and resolution = 2 × 4 × 6 mm) was used to obtain the experimental B_1_^+^ transmit field maps with the cylindrical phantom. The flip angle (*α*) with short TR_1_ and TR_2_ was calculated by, $\alpha =\arccos\frac{rn-1}{n-r}$, where n = TR_2_/TR_1_ and $r\approx \frac{1+n\;cos\alpha }{n+cos\alpha }$ [35]. And it converted to B_1_^+^ with $\alpha =2\pi \gamma B_{1}^{+}\tau$, where *γ* is the gyromagnetic ratio (42.57 MHzT·^−1^) and *τ* is the width in seconds of the RF pulse [36].

## III. Results

### A. Comparison of Coupling and Noise Covariance

As indicated in Table I, the 8-channel MDH array showed improved decoupling (*S*_21_) compared to the monopole and dipole antenna arrays. The values of the monopole antenna array were presented in Woo *et al.* [24]. The coupling between adjacent channels was in the range of −8.6 dB to −9 dB for the 8-channel monopole antenna array, −9.7 dB to −12.5 dB for the 8-channel dipole antenna array, and −13.4 dB to −21 dB for the 8-channel MDH array. The decoupling values of arrays were achieved in the absence of any decoupling circuit.

**Fig. 3.**
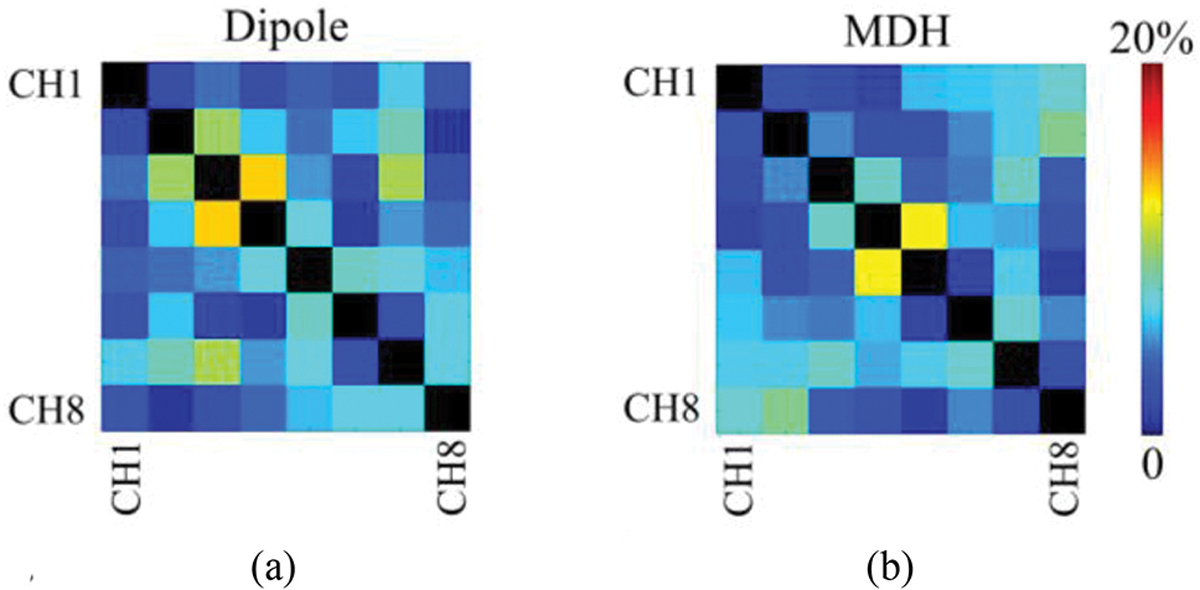
Noise covariance matrices of the 8-channel dipole (a) and MDH (b) arrays.

A comparison of the 8-channel dipole [Fig. 3(a)] and MDH [Fig. 3(b)] array indicated that the values for noise covariance between adjacent channels of the 8-channel MDH array (mean: 0.06, min: 0.01, and max: 0.12) were overall lower when compared to the 8-channel dipole antenna array (mean: 0.07, min: 0.02, and max: 0.14).

### B. Performance Comparison With a Phantom and a Human Model

**Fig. 4.**
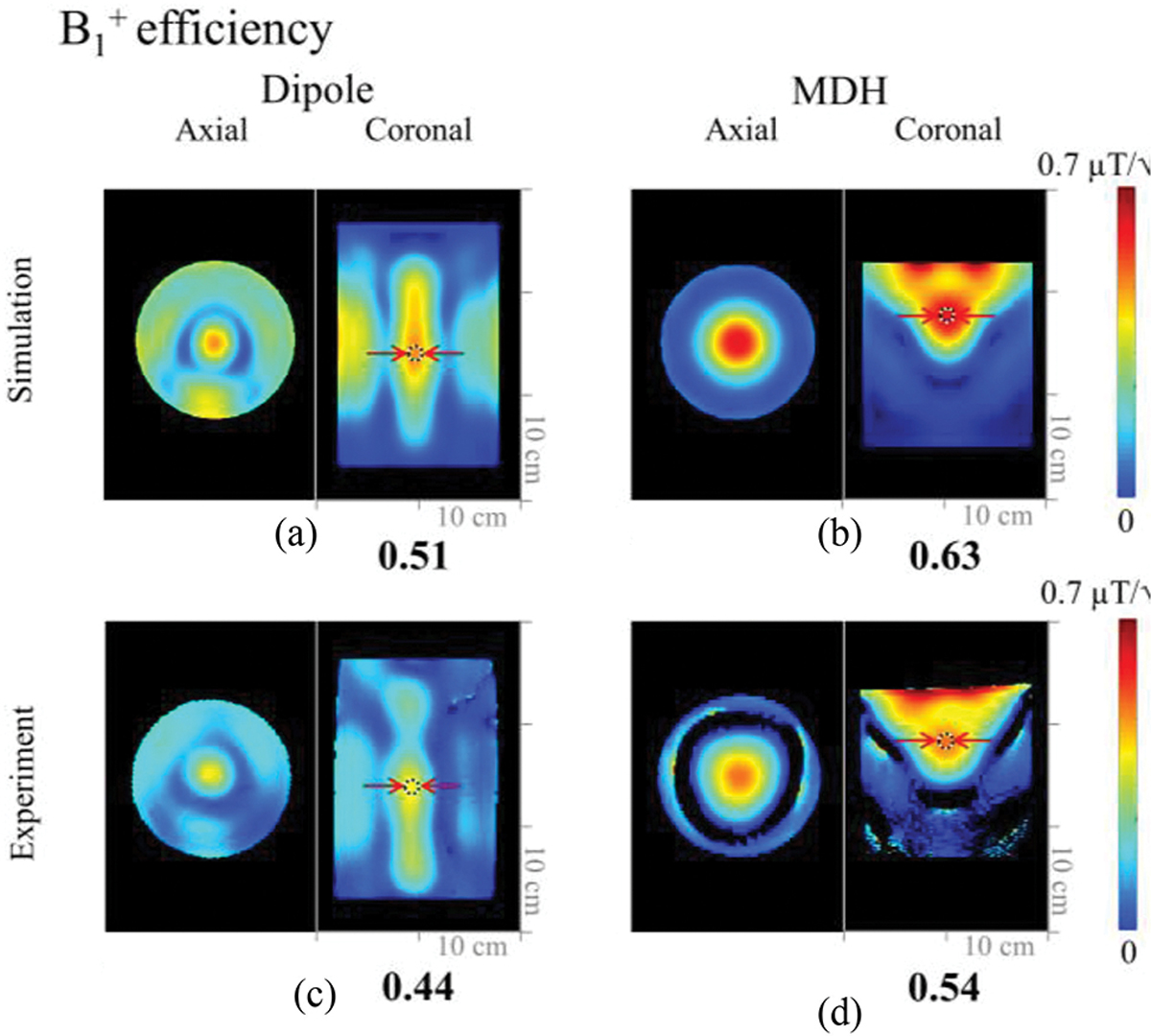
Simulated (a) and (b) and experimental (c) and (d) data sets for the 8-channel dipole and MDH arrays in the axial and coronal planes. Red arrows indicate ROIs where values are measured and compared.

**Fig. 5.**
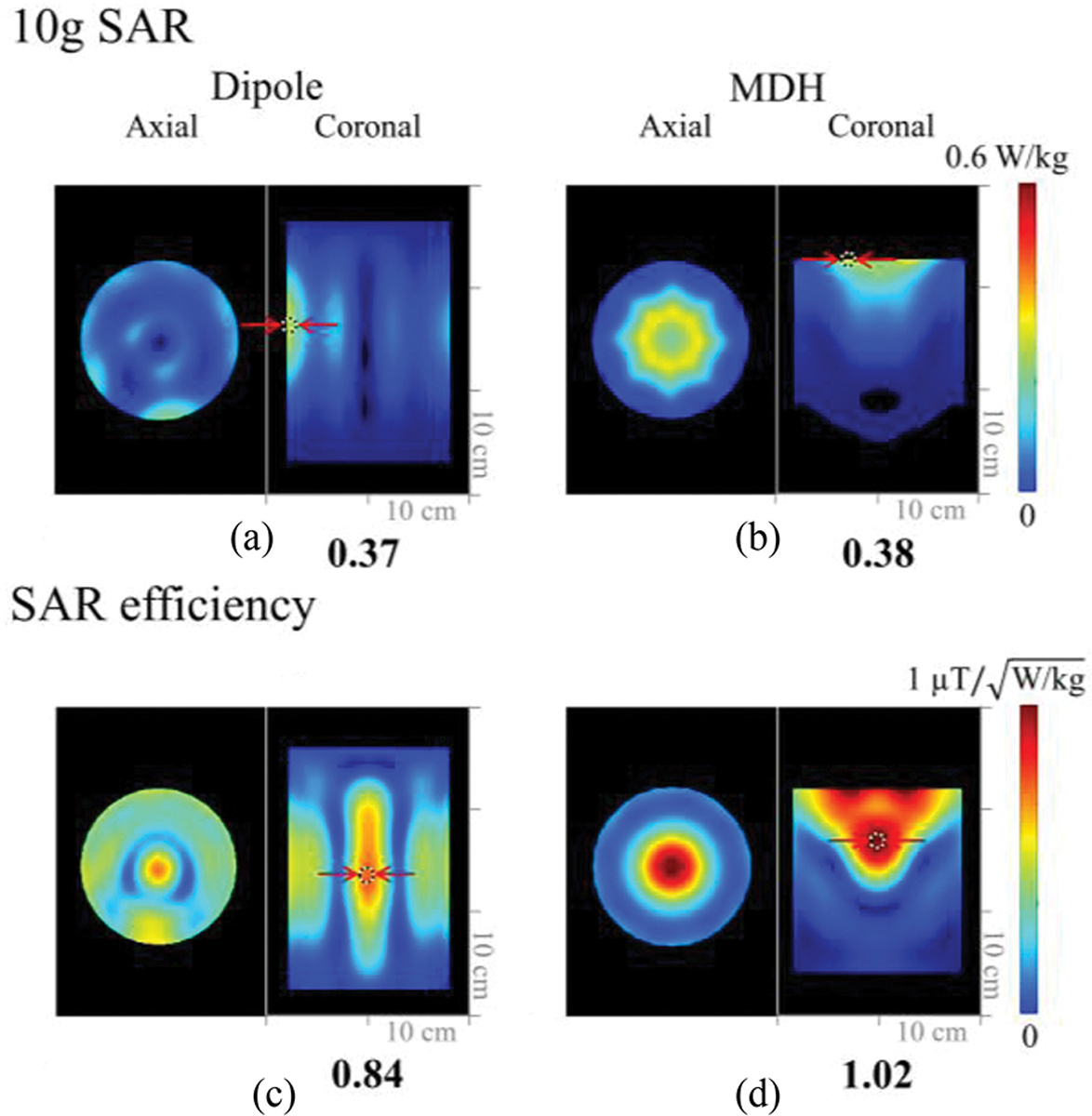
10 g SAR (a) and (b) and SAR efficiency (c) and (d) of the 8-channel dipole and MDH arrays in the axial and coronal planes.

Fig. 4 shows the B_1_^+^ efficiency maps that were obtained in both simulation 4(a) and (b) and experiments 4(c) and (d) with a phantom. The simulated and experimental B_1_^+^ efficiency of the 8-channel MDH array showed approximately a 19% higher value within the indicated ROI in the phantom over the 8-channel dipole antenna array. As shown in Fig. 5(a) and (b), the peak 10 g SAR values were similar for the 8-channel dipole (0.37 W/kg) and MDH (0.38 W/kg) arrays. In terms of SAR efficiency, the simulation showed that the 8-channel MDH array [Fig. 5(d)] had an 18% higher value compared to the 8-channel dipole antenna array [Fig. 5(c)].

**Fig. 6.**
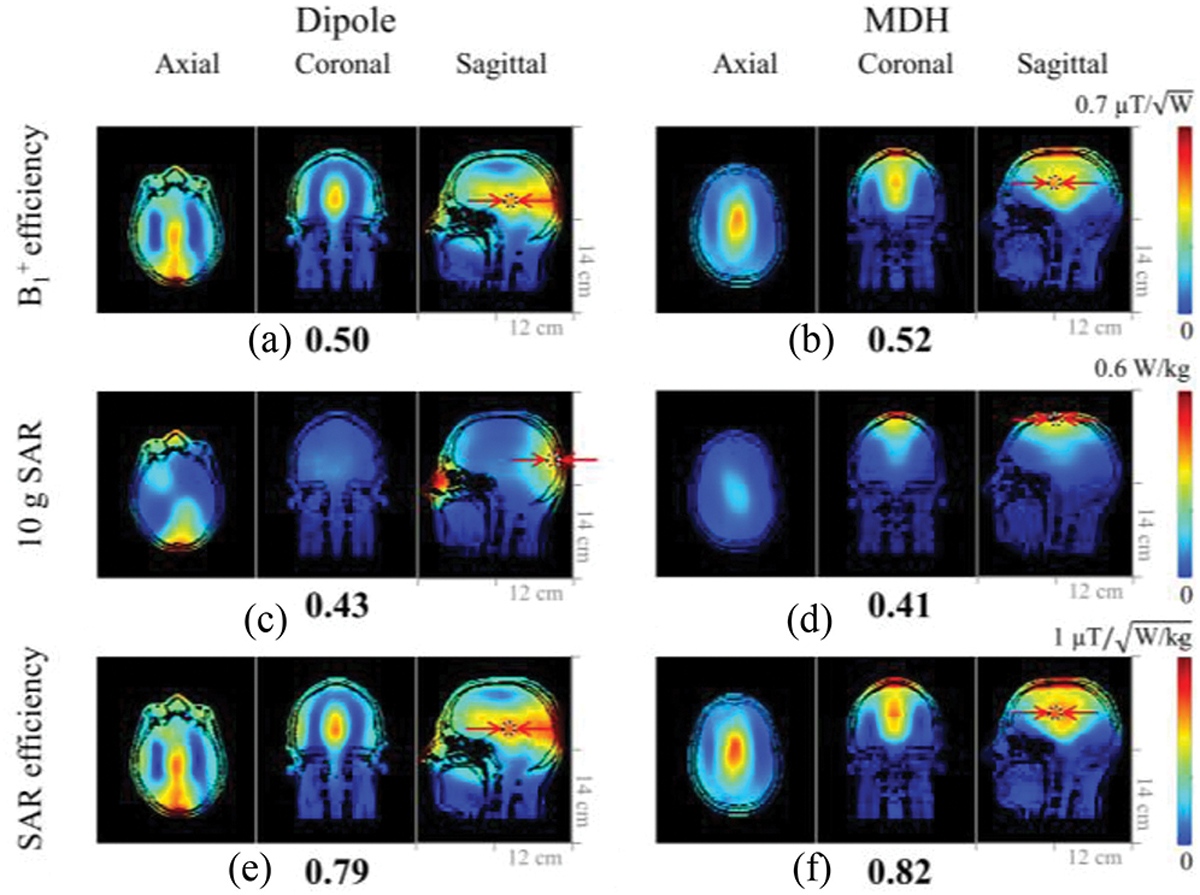
Simulated B_1_^+^ efficiency, 10 g SAR, SAR efficiency maps of the 8-channel dipole (a), (c), and (e) and MDH (b), (d), and (f) arrays. Fig. 6(b) in the sagittal view and Fig. 1(d) are the same figures with a different scale (1.0 and 0.7 μT/√(W)).

Fig. 6 shows B_1_^+^ efficiency, 10 g SAR, SAR efficiency, and overall coverage for of each array with a human model. The overall values between the dipole and the MDH arrays were found to be similar while the coverage differed as expected. The straight dipole antenna array demonstrates improved coverage across the lower and central brain regions, whereas the MDH array excels in the upper brain regions. However, the MDH array showed slightly better overall B_1_^+^ and SAR efficiency compared to the dipole antenna array.

## IV. Discussion

We designed and evaluated a novel 8-channel mono-dipole hybrid head array and compared it to performance values achieved with a monopole antenna array from our previous publication [24]. A summary of *S*-parameters (*S*_11_ and *S*_21_), peak 10 g SAR, and SAR efficiency of the dipole antenna and MDH arrays can be seen in Table I. Improved *S*-parameters were achieved with the MDH array compared to both monopole and dipole antenna arrays. We attribute the reduced interference between the channels of the 8-channel MDH array to its asymmetric feed structure [37], [38]. This asymmetric structure of the MDH array causes a change in the current distribution, as well as reduced magnetic flux coupling and noise covariance among the channels.

The deliberate low channel count of the 8-channel arrays described in this study supports sufficient array decoupling without further decoupling circuitry, and represents a valuable validation step, but does not allow to fully exploring possible limitations of the different array types for higher channel count. We anticipate that the effectiveness of the common ground of the MDH concept would possibly be improved with higher number of channels, and that more antenna elements could be expected to produce a more effective ground plane. However, the associated E-field distribution would need to be carefully evaluated.

Comparing the dipole and the MDH array with the phantom, we observed significantly improved B_1_^+^ and SAR efficiency in the upper regions. However, these improvements were more modest in the human head model. A possible explanation for this is that for the chosen circular array geometry the distance between the antenna array elements and the phantom is equidistant, while for the human head model, nonuniform distances between the antennas to the human subject are observed. Thus, it is expected that resulting B- and E-fields will differ between a human model and a phantom [15], [24].

When comparing overall coverage of the dipole antenna array with the MDH array, the MDH produced smaller dense B_1_^+^ field profile within the phantom [Fig. 4(b) and (d)], whereas in the case of the human model, the dipole antenna array [Fig. 6(a)] showed the better coverage of the lower and central brain and produced a broader B_1_^+^ field pattern than the MDH array. However, in the superior part of the brain, the MDH array [Fig. 6(b)] achieved significant higher B_1_^+^ efficiency.

## V. Conclusion

We proposed, built, and evaluated a hybrid array of MDH array for UHF brain imaging at 447 MHz/10.5 T. The main advantage of the MDH array is significantly higher performance in the upper part of the brain and improved decoupling among neighboring channels. This can potentially support either tighter channel spacing or increased antenna channel density, or interleaved arrays using, for example, sleeve antennas [24], [38]. We anticipate that such interleaved arrays can extend the coverage toward the central brain region thus addressing a current weakness of the MDH array which is more limited whole brain coverage compared to dipole or loop arrays. Besides extended coverage, in future coil designs we also plan to increase the number of array channels and evaluate the B_1_^+^ and SAR efficiency of this denser interleaved array in simulation and eventually in-vivo in a human head UHF MRI.

## Figures and Tables

**TABLE I:** Measured *S*-Parameters, Peak 10 g SAR, and SAR Efficiency of the 8-Channel Monopole*, Dipole and MDH Arrays, Respectively

	Monopole	Dipole	MDH

S_11_ (dB)	Min: −9	Min: −14.2	Min: −16.4
- Reflection	Max: −11.2	Max: −29.8	Max: −32.3
S_21_ (dB)	Min: −8.6	Min: −9.7	Min: −13.4
- Coupling	Max: −9	Max: −12.5	Max: −21
Peak 10 g SAR (W/kg)	0.74	0.43	0.41
SAR efficiency	0.83	0.79	0.82

The Values of the Monopole Antenna Array Were Exported From Woo *et al*. [24]
